# Meta‐inflammaging at the crossroad of geroscience

**DOI:** 10.1002/agm2.12078

**Published:** 2019-09-08

**Authors:** Guobing Chen, Raymond Yung

**Affiliations:** ^1^ Institute of Geriatric Immunology School of Medicine Jinan University Guangzhou China; ^2^ Department of Microbiology and Immunology School of Medicine Jinan University Guangzhou China; ^3^ Geriatrics Center and Institute of Gerontology University of Michigan Ann Arbor MI USA; ^4^ VA Ann Arbor Geriatrics Research, Education and Clinical Center Ann Arbor MI USA; ^5^ Department of Internal Medicine Division of Geriatric and Palliative Medicine University of Michigan Ann Arbor MI USA

**Keywords:** geroscience, inflammaging, metabolic inflammation

## Abstract

Geroscience posits that selected fundamental biological processes are the foundation of age‐related chronic diseases and are responsible for the decline in physical and mental function in old age. Late‐life chronic low‐grade inflammation (“inflammaging”) and altered signal transduction pathways in metabolism have been identified as two of the key themes in the aging process. Age‐related changes in the immune and metabolic responses are also recognized as playing a critical pathogenic role in most common chronic medical conditions that plague the elderly. Emerging investigations emphasize the interconnectedness of the immune and metabolic responses in aging, an area of gerontological research that can be termed “meta‐inflammaging.”

## 1 INTRODUCTION

The concept of “geroscience”^1^ represents a fresh perspective in our understanding of the molecular underpinning of aging, and offers a roadmap for future investigation into the role that selected critical biological processes (adaptation to stress or resilience; epigenetics; inflammation; macromolecular damage; metabolism; proteostasis; and stem cells and regeneration) play in explaining the high prevalence of chronic diseases in old age. Biomedical and gerontological research has traditionally relied on the reductionist approach to identifying single biochemical pathways and molecular target(s) for therapeutics. However, the field of geroscience emphasizes the interconnectedness and interdependency of key biological processes in aging. While seven biological processes are currently regarded as the underpinning of the aging process, the degree and rate of change of the different biological processes are expected to be unique in different individuals. Additionally, future investigations may result in the addition of other novel biological areas to the current geroscience “pillars.” Because of their interconnectedness, age‐related dysfunction in one geroscience area will likely influence other critical biological processes in geroscience. This in turn may result in varying degrees of susceptibility to the development of the multiple chronic conditions prevalent in late‐life. Conversely, future therapies that target one geroscience process will likely have a positive impact on the other interconnecting pillars of geroscience, even if the benefits are viewed as “off‐target” effects. The current review will focus on discussing our evolving understanding of the link between chronic low‐grade sterile inflammation prevalent in old age and obesity‐associated metabolic inflammation, as an illustration of the important link between the geroscience pillars.

## 2 INFLAMMAGING

The immune system undergoes significant changes during development and after midlife. This has broad implications and may help explain why older adults are more susceptible to diverse illnesses, including infectious disease, cancer, and autoimmunity. Extensive research over the past two decades has confirmed early observations that old age in mammals, particularly elderly adults with poor physical function or frailty, is linked to a state of chronic low‐grade sterile inflammation (inflammaging) with elevated pro‐inflammatory cytokines, such as interleukin (IL)‐6 and tumor necrosis factor (TNF)‐α.^2^, ^3^ Importantly, interventions that enhance life span and health span generally reduce the systemic low‐grade inflammation in old age. Per geroscience, inflammaging is one of seven evolutionarily conserved mechanistic pillars of aging that are shared by many aging‐related conditions.

Inflammaging is considered to be an evolution‐conserved, dynamic, progressive, systemic, and adaptive consequence of protective response in our body. Age‐related increases in pro‐inflammatory and decreases in anti‐inflammatory factors have been documented in both invertebrates and vertebrates. Interestingly, a set of chemokines was also found to be increased in elderly humans and animals,^4^, ^5^, ^6^, ^7^ indicating a broad level of inflammation throughout the body's immune system.^8^

The source of the inflammatory cytokines is controversial. The inflammatory factors are produced not just by immune cells, but also non‐immune cells, especially adipocytes^9^ and senescent cells.^10^ The immune cells, mainly innate immune cells, particularly macrophages, are considered the major inflammatory cytokine producers. The activation of immune responses is presumably triggered by exogenous and endogenous danger signals. Chronic and/or repeated bacterial and viral infections, such as cytomegalovirus latent infection and gut microbiota dysbiosis,^11^, ^12^, ^13^ are considered the important triggers to activate immune cells in the elderly. Another exogenous trigger is the so‐called “molecular garbage,”^14^ which is produced from cell debris or misplaced molecules from the aged cells, organs, and systems. One of the critical functions of the immune system is immune surveillance: the recognition and clearing of the exhausted, senescent, mutated, or shattered cellular or molecular garbage in the circulation and local tissues. With the decline in immune function during aging, this overaccumulated garbage cannot be cleared sufficiently, resulting in a persistently activated immune system and consequent inflammation.

Similar to cells in other organs and systems, immune cells also become senescent or exhausted during aging, which in turn also contributes to systemic low‐grade sterile inflammation in old age. The reported endogenous triggers include mitochondrial damage‐associated molecular patterns (DAMPs; eg, cardiolipin and mtDNA), reactive oxygen species (ROS), nuclear DNA damage, telomere shortening, spindle stress, and oncogene activation.^15^, ^16^, ^17^ More sources of inflammaging include environmental exposures (such as pollution and cigarette smoking over one's life span), over‐activated coagulation system, impaired regulation of complement pathway, and altered macromolecule modification (such as galactosylated N‐glycans).^18^, ^19^, ^20^, ^21^, ^22^

The exogenous and endogenous threats can be clarified as pathogen‐associated molecular patterns (PAMPs) and DAMPs that are recognized by different pattern‐recognition receptors (PPRs).^23^ These PPRs include antigen receptor (B‐cell and T‐cell receptors, B‐cell receptor/Immunoglobulin and T‐cell receptor) and Toll‐like receptors (TLR) on the cell surface,^24^, ^25^ and the cytosolic receptors, such as NOD‐like receptors (NLRs), RIG‐I‐like receptors (RIRs), aryl hydrocarbon receptor (AhR), and cytosolic DNA sensors (CDSs; which work through stimulator of IFN genes [STING]).^26^, ^27^ Age‐associated increased exposure to PAMPs (such as latent viruses, gut microbiota dysbiosis, or the endogenous macromolecular garbage) mimic antigen epitope pattern and activate either cell surface or cytosolic receptors persistently to promote expression of inflammatory cytokine genes. DAMPs mainly activate cytosolic sensors to regulate inflammatory cytokines gene expression directly or result in epigenetic drift resetting the “immunological rheostat” of immune cell cytokine production machinery. The epigenetic drift in aging primarily involves alteration (both increased and decreased) of DNA methylation and modification of histone and chromatin structure.^28^, ^29^, ^30^

The decrease in naive B and T lymphocytes and antigen receptor repertoire impair the clearance capability of exogenous antigens and senescence cells.^31^, ^32^, ^33^, ^34^ Although thymic‐derived naturally regulatory T cells (nTregs) are increased, both CD4‐ and CD8‐inducible Treg (iTreg) cells decline significantly with age.^35^, ^36^ It has been suggested that increased nTreg is correlated with aging‐associated high susceptibility for cancer, infectious diseases, and neurodegenerative diseases. However, the roles of Treg, especially iTreg in inflammaging, are incompletely understood. The conceptual conflict between increased Treg and inflammaging is potentially explained by: (a) less repressive function of nTreg, even though the number is increased (although others have shown that not to be the case^36^); (b) impaired recruitment to the inflammatory sites; (c) more memory Treg specific for selected antigens, but not to those that are involved in downregulating inflammation; and (d) long survival capability of aged Treg because of downregulation of pro‐apoptotic molecule Bim.^37^ More investigations are required to clarify the roles of physiological immune suppression in inflammaging.

## 3 META‐INFLAMMAGING

Another potential source of pro‐inflammatory cytokines in aging is adipose, or fat tissue, which can be the largest organ in obese individuals. Obesity has become a global epidemic in males and females of all racial groups, including more recently in low‐ and middle‐income countries. Excessive weight gain accelerates, and weight loss prevents, the onset of many common illnesses in old age.^9^ Aging alters adipose tissue distribution, composition, and function, in part as a consequence of the sex hormone changes that occur in midlife and beyond. The overall effects include increase in abdominal fat and redistribution of subcutaneous fat to the visceral and bone marrow fat depots, decrease in brown fat activity, and impaired thermogenic capacity.^9^, ^38^ On the surface, it is difficult to envision an evolutionary advantage of an organism developing chronic low‐level metabolic inflammation in old age. However, longevity, at least viewed through the lens of modern humans and an evolutionary perspective, is a relatively new phenomenon. Interestingly, it has been hypothesized that mitochondrial uncoupling of substrate oxidation from ADP phosphorylation may protect cells from conditions favoring ROS production, which may be advantageous in aging and extend life span.^39^

Obesity has been linked to a low‐grade sterile chronic inflammatory state, with similarities to inflammaging, and is termed “meta‐inflammation.”^40^, ^41^ There remain important unanswered research questions regarding the relationship between inflammaging and meta‐inflammation in old age, including whether they share the same source of the associated pro‐inflammatory cytokines. It is unclear how immune senescence, generally viewed as a decline in immune responses in old age, interacts with meta‐inflammation in age‐related obesity (meta‐inflammaging) to influence acute/chronic disease susceptibility and outcomes.

The “obesity paradox”^42^ describes the phenomenon that overweight older adults live longer and have better outcome (resilience) from stressful events than slender persons with similar ailments, such as in patients undergoing percutaneous coronary artery intervention for cardiovascular diseases. While using body mass index (BMI) is not ideal for measuring obesity, existing data would indicate that the mortality risk is lowest in individuals with BMI around 25 kg/m^2^. Thus, being exceptionally thin should not be a desirable trait in old age, and a modest amount of adipose tissue may be important to maintain resilience in older and frail adults. Whether to treat, and how to treat, obesity (particularly those who are moderately overweight) in old age is also controversial, as there is a paucity of data to guide such therapy or even validate its benefit.

Nutrient sensing/intake are tightly linked to the body's immune and inflammation responses. There is tantalizing evidence that the immune/inflammatory system may have coevolved with the endocrine/metabolic system.^2^, ^41^ For example, macrophages play a critical role in adipose tissue inflammation and also share functional similarities with adipocytes/pre‐adipocytes, including their responses to bacterial products. Sterile water and nutrition, and hand hygiene, are all relatively new modern phenomena, and the immune system in the gut has to adapt to hordes of resident and external microbes regularly. Interestingly, even normal meals can result in a post‐prandial inflammatory cytokine surge.^41^ There is evidence from studies of lower organisms that the immune and metabolic systems may have evolved and shared an ancestral structure.^41^

Obesity research has traditionally focused on the idea that there is a perturbed balance between energy intake and expenditure, often utilizing animals fed with a high‐fat diet. The obesity phenotype observed in mid‐ and late‐life is often regarded as resulting from a reduction in basal metabolic rate and reduced energy expenditure. White visceral and gonadal adipose tissue from obese individuals and animals produces higher levels of pro‐inflammatory cytokines, such as TNF‐α, which may directly affect glucose tolerance and insulin sensitivity.^41^, ^43^ Similar to diet‐induced obesity, macrophages from old animals fed a “normal” (non‐high‐fat) diet exhibit a shift from the “resident” M2 to pro‐inflammatory M1 cytokine profile.^44^ The observed macrophage inflammation in old adipose tissue is linked to elevated endoplasmic reticulum (ER) stress (adaptation to stress), and reduction in ER stress causes a reduction in adipose tissue inflammation,^45^ highlighting the link between the two pillars (inflammation and adaptation to stress) of aging in geroscience. Furthermore, adipose tissue from old animals has perturbed autophagy function (proteostasis), which is also linked to old age ER stress response and inflammation.^46^ Interestingly, a similar link between ER stress response and adipose tissue inflammation has also been reported in diet‐induced obesity.^47^

Despite many similarities between diet‐ and age‐related obesity, there are also important differences, in part due to the compounding effects of immune senescence, distinguishing metabolic inflammation from diet‐induced obesity and aging‐associated meta‐inflammaging. In old adipose tissue, there is an accumulation of senescent cells that secrete pro‐inflammatory cytokines, including IL‐6, IL‐8, and TNF‐α, collectively known as the “senescence‐associated secretory phenotype.”^48^, ^49^ Senescent cells accumulate with age, in part due to impaired clearance mechanism,^50^, ^51^ which may contribute to the observed meta‐inflammaging. Reducing senescent cell burden in obese mice has recently been linked to improved glucose tolerance, enhanced insulin sensitivity, less accumulation of macrophages, and reduced pro‐inflammatory cytokine production.^52^

While adipose dysfunction does not solely rely on immune‐cell‐driven inflammation, adipocyte function also cross‐talks with immune cells and independently contributes inflammatory signals.^9^ Aging is also linked to an accumulation of regulatory T cells (Tregs) that is related to age‐related epigenetic drift and T‐cell hypomethylation.^36^ The consequent suppression of T‐cell responses has been linked to immune senescence and may contribute to the increase in susceptibility to age‐related chronic diseases. Obese mice and humans with diabetes have increased Th17 cells that contribute to a decreased number of Tregs.^53^ Depletion of fat‐specific Tregs has been found to prevent age‐related insulin‐resistance,^54^ confirming the interplays between immune senescence and meta‐inflammation.

## 4 CONCLUSION

Geroscience represents an important advance in defining the conceptual framework for biogerontology. Future advances will likely lead to further refinement of our understanding of the interconnecting geroscience themes. Adiposity has emerged as a major source of inflammation in both diet‐ and aging‐associated obesity, which in turn is linked to most common diseases in old age. Pathways involved in meta‐inflammaging should therefore be attractive targets for future research to modulate the aging process and increase the life span and health span.

## AUTHOR CONTRIBUTIONS

G.C. and R.Y. designed the topic and wrote the paper.

## CONFLICTS OF INTEREST

The authors have no conflicts of interest to declare related to the content of the article.
